# Influences of COMT and 5-HTTLPR Polymorphisms on Cognitive Flexibility in Healthy Women: Inhibition of Prepotent Responses and Memory Updating

**DOI:** 10.1371/journal.pone.0085506

**Published:** 2014-01-20

**Authors:** Elisabeth M. Weiss, Günter Schulter, Andreas Fink, Eva M. Reiser, Erich Mittenecker, Harald Niederstätter, Simone Nagl, Walther Parson, Ilona Papousek

**Affiliations:** 1 Department of Psychology, Biological Psychology Unit, University of Graz, Graz, Austria; 2 Institute of Legal Medicine, Innsbruck Medical University, Innsbruck, Austria; Radboud University, Netherlands

## Abstract

Understanding genetic factors that affect monoamine neurotransmitters flux in prefrontal cortex may help to further specify the complex neurobiological processes that underlie cognitive function and dysfunction in health and illness. The current study examined the associations between the polymorphisms of dopaminergic (COMT Met158Val) and serotoninergic (5-HTTLPR) genes and the sequential pattern of responses in a motor random generation task providing well-established indexes for executive functioning in a large sample of 255 healthy women. Participants homozygous for the Met allele of the COMT polymorphism showed impaired inhibition of prepotent responses, whereas individuals homozygous for the s-allele of the 5-HTTLPR showed a restricted ability to update information in working memory. Taken together the results indicate differentiated influences of dopaminergic and serotonergic genes on important and definite executive sub-processes related to cognitive flexibility.

## Introduction

Most psychiatric disorders including affective disorders, psychotic problems or addiction have been linked to executive dysfunction such as impairment in cognitive flexibility [1]. A large body of evidence supports the role of the dopaminergic system in modulating executive functions [2], but recent research also indicated a role of the serotoninergic system [3]. Evidence of high heritability of executive functioning [4] has stimulated research on specific genetic variants influencing executive functions. However, results have been heterogeneous, and the mechanisms by which dopamine- and serotonin-related genetic variants influence cognitive processes are still poorly understood.

The Catechol-O-methyltransferase (COMT) enzyme plays an important role in modulating the dopamine levels in prefrontal cortex [5]–[7]. A common functional polymorphism at codon 158 in the gene on chromosome 22q11 results in a substitution of valine (Val) by methionine (Met) in the peptide sequence (Val158Met), that affects the thermostability of the enzyme and leads to a 3–4 times lower enzymatic activity in Met carriers [5]; [8]. Thus, homozygotes for the low activity Met-allele are expected to have higher prefrontal dopamine levels, whereas individuals with homozygosity for the high activity Val-allele are expected to have lower levels of endogenous dopamine. Because of the relevance of the COMT 158 polymorphism in prefrontal dopamine catabolism, a number of studies have explored this single nucleotide polymorphism as a candidate gene for schizophrenia, Parkinson’s disease, ADHD and other complex psychiatric disorders associated with executive dysfunctioning [9]–[15]. However, meta-analyses on COMT effects on cognitive functioning have reported mixed results [16]–[17], possibly due to methodological differences between studies such as executive sub-processes being studied. More recent studies suggested that the Met allele enhancing tonic dopamine might stabilize and help maintain relevant information while the Val allele enhancing phasic dopamine might be important for updating and cognitive flexibility [18]–[20].

It is widely accepted that serotonin (5-hydroxytryptamine, 5-HT) plays a central role in the regulation of mood and emotion but emerging evidence also suggests a role of 5-HT in executive functions such as top-down attention and inhibitory control (for review see [21]). An important regulator of 5-HT function is the serotonin transporter (5-HTT) which mediates the intracellular reuptake of released serotonin and modulates the concentration of serotonin in extracellular fluids. A polymorphism of the serotonin transporter linked promoter region (5-HTTLPR) polymorphism results in two allelic variants differing in length: a short (s) allele and a long (l) allele. The presence of one or two short (s) alleles is associated with reduced transcriptional efficiency and significant decreases in serotonin reuptake compared to the long (l) allele [22]–[23]. While the short allele of the 5-HTTLPR genotype is associated with emotionality or stress sensitivity and has been typically studied as a vulnerability factor for depression and anxiety-related disorders [21] recent research also suggest a potential influence of 5-HTTLPR on cognitive functioning, especially on executive function [24]–[27] sustained attention [27], and decision making e.g. [28] with most studies showing better performance in individuals possessing the short allele of the polymorphism compared to the long allele. However, results have been inconsistent with some authors (e.g. [29]) showing significantly reduced cognitive flexibility, as measured by object discrimination reversal learning (ODRL) and related tasks such as extinction of an instrumental response in Rhesus monkeys homozygous for the s allele (s/s) compared with monkeys with either one or two copies of the l allele. Particularly, gender may moderate the effects of 5-HTTLPR on neurocognition and in a study by Price et al., [30] the 5-HTTLPR s-allele was associated with impaired figural memory performance and larger left hippocampal volume only in young adult women but not in men.

Early work linking genes to cognitive functions typically relied on standard neuropsychological tests of executive functions such as the Wisconsin Card Sorting Test (WCST), which is a diffuse indicator of brain function comprising several cognitive components. Therefore, more sensitive neuropsychological tools that parse and segregate underlying cognitive processes are warranted to move the research on genetic individual differences in executive functioning forward. It has been posited that some executive functions are difficult to quantify experimentally, because they are rooted in the temporal domain [31]. This is particularly typical of measures of cognitive flexibility which refers to the function of dynamically activating and modifying cognitive processes in response to changing conditions and demands. Among the most frequently postulated executive functions that can be subsumed under the concept of cognitive flexibility are the inhibition of dominant or prepotent responses and memory updating, which are moderately but clearly separable functions [32]. Inhibition refers to the ability to inhibit or override the tendency to produce a more dominant or automatic response when necessary for achieving the current behavioral goal. Updating refers to the ability to monitor incoming information and to adjust the content of working memory according to the current behavioral goal [32].

The examination of the temporal organization of behavior requires a behavioral paradigm that enables the repeated measurement of many observations of behavior. Paulus and co-workers [31] used a two-choice guessing task to obtain sequences of behavior and a measure of entropy to quantify the degree to which the past behavior predicted the current behavior. It was demonstrated that the current behavior of schizophrenia patients was more influenced by previous responses than that of healthy individuals [33]–[34]. Predictability (non-randomness) as measured by entropy was related to impaired executive functioning (more perseverative responses) on the Wisconsin Card Sorting Test [31].

The generation of random sequences, also relying on the sequential pattern of responses, may be another promising tool in this context. Random motor generation tasks have proved to be a useful diagnostic tool for the identification of clinically relevant impairments of executive functions in psychiatric and neurological disorders such as schizophrenia [34]–[38] and Parkinson’s disease [39]–[41]. But there is also substantial interindividual variability in the randomness of produced sequences in healthy individuals that is determined by the efficiency of executive processes [42]–[46].

The major advantage of random sequence generation tasks is that performance essentially relies on the two components of cognitive flexibility mentioned above [32]; [47]; [48]: (a) the inhibition of prepotent responses (including the inhibition of developing routines and the suppression of overlearned response stereotypes) and (b) memory monitoring and updating. Most important, using a random motor generation task such as the Mittenecker Pointing Test (MPT) and specific measures of randomization performance (based on information theory analysis), the inhibition component and the memory component can be separated and therefore independently studied using only one simple test [48]. Furthermore, unlike random number or letter generation, in the MPT the suppression of overlearned response stereotypes (i.e., the inhibition of sequences that have been overlearned through years of experience such as counting up or down or producing letters in alphabetical order) does not apply. Consequently, one of the measures of the MPT, the context redundancy (CR) reflects the efficiency of a specific inhibitory process, that is, the capability to inhibit developing routines (the naturally occurring tendency to repeat already selected sequences). The symbol redundancy (SR) reflects the efficiency of memory monitoring and updating, that is, the capability to hold information in memory in order to monitor the distribution of chosen responses and to continually update it [32]; [48].

It has been proposed that the sequential pattern of behavior in terms of interdependence of responding may be closely linked to the functional status of the dopamine system [49]. Dopaminergic stimulation produced perseverative responding and stereotyped behavior [50]. Repetitive/stereotyped behavior has also been linked to low levels of brain serotonin, local depletion of serotonin in the prefrontal cortex, for instance, increased perseverative behavior in monkeys [51]–[52]. In addition, both dopaminergic (e.g., [10]; [53]–[54]) and serotonergic neurotransmission [26] is involved in working memory functions. Important in the context of the motor random generation task, findings have suggested that prefrontal serotonergic activity is particularly related to the organization of working memory that relies on one’s own behavioral responses (rather than on external sensory information; [55]). Overall, few studies simultaneously examined the relationship between polymorphisms of dopaminergic and serotoninergic genes to cognitive functions in healthy subjects in order to test their specific relevance.

The purpose of this study was to assess the association between the polymorphisms of dopamine and serotonin systems’ genes and the generation of random sequences using the Mittenecker Pointing Test, which provides specific and well-established indexes of executive functioning and cognitive flexibility. We expected the Met allel of the COMT polymorphism, and the short allele of the 5-HTTLPR genotype, to be associated with relatively poorer performance on the inhibition and the memory updating component of cognitive flexibility, respectively.

## Methods

### Ethics Statement

The study was performed in accordance with the 1964 Declaration of Helsinki and was approved by the Ethics Committee of the Karl-Franzens University, Graz. After complete description of the study, written informed consent was obtained from all participants.

### Participants

A total of 255 healthy female students aged 18 to 59 years (*M* = 22.7, *SD* = 4.8) were recruited from a larger screening sample (n = 624). All participants were Caucasian and native German speakers recruited at the local university. The students received course credit for their participation. No participant reported suffering from neuropsychiatric disease or was using psychoactive medication. They completed a screening questionnaire concerning demographic data and general information. Furthermore, all subjects were tested with the Mittenecker Pointing Test.

Buccal swabs were taken to genotype all participants for the 5-HTTLPR and COMT Met158Val polymorphisms. Data of the total screening sample were in Hardy-Weinberg equilibrium (COMT X^2^(n = 624,df = 1) = 1.50, ns.; 5-HTTLPR X^2^(n = 624,df = 1) = 0.62, ns.). Table 1 shows the distribution of COMT and 5-HTTLPR genotypes. Table 1 shows the distribution of COMT and 5-HTTLPR genotypes.

**Table 1:** Distribution of COMT and 5-HTTLPR genotypes.

		COMT			
		Val/Val	Val/Met	Met/Met	
5-HTTLPR	s/s	11	30	18	59
	s/l	19	55	32	106
	l/l	21	39	30	90
		51	124	80	

### Mittenecker Pointing Test (MPT)

The MPT [48] is a computer-based test in which the participants are instructed to press the keys of a keyboard with nine unlabeled keys irregularly distributed over the board with their index finger in the most random or chaotic order possible. The responses were paced by an acoustic signal (1/sec.) to control the rate of production. A total of 180 responses were required. Two quantitative measures of deviation from randomness, based on information theory analysis were used [36]; [48]; [56]. They are based on the principle that if information is maximal, redundancy is minimal, and the series approximates randomness (i.e., maximal disorder).

The symbol redundancy (SR) refers to the inequality of the relative frequencies of chosen keys. The randomness of a series decreases with increasing deviation of the frequency distribution of chosen response alternatives from equality. A SR score of zero denotes maximal equality of the relative frequencies and thus minimal predictability, whereas a score of 1.000 denotes maximal redundancy and, thus, a lack of randomness. SR is related to the memory component (memory monitoring/updating) of random sequence generation [32]; [48], and is equivalent to what in the literature is sometimes termed R score.

The context redundancy (CR) is based on the sequential probability of each chosen key. In true random series all possible dyads (pairs of adjacent responses), but also all possible triples, quadruples etc. are approximately equiprobable, whereas the frequency distribution of dyads deviates from equality, if responses are continuously influenced by previously chosen alternatives. As in most studies of response series, in this paper, too, the CR measure (“second order CR”) is based only on the distribution of pairs of adjacent responses and of single responses as a sufficient measure of redundancy of response series. (However, evidence for interesting results when evaluating higher order dependencies is given, e.g. in [57]–[58]).

The major part of the interindividual variance in CR is due to the tendency to repeat certain sequences of responses en bloc [36]. A CR score of zero denotes the complete absence of any regular pattern, a score of 1.000 denotes the presence of a fixed, repetitive response pattern and thus a lack of randomness. The CR corresponds to the so-called random number generation score [59]. In the MPT, it reflects the inhibition of developing routines in random sequence generation ([32]. For detailed instructions for the MPT and information how to compute SR and CR see [48]).

### Genotyping

#### DNA extraction

DNA was extracted from individual saliva swabs using a modified Chelex method. 1.25 ml of a solution containing 5% Chelex (Biorad, Hercules, CA, USA) and 200 µg/mL Proteinase K (Roche, Mannheim, Germany) were added to the saliva swab placed in a 1.5-ml microcentrifuge tube. The solution was incubated at 55°C for 30 min, vortexed, incubated at 90°C for 8 min, and finally centrifuged at 13,000×g for 5 min.

#### Genotyping of 5-HTTLPR

Polymerase chain reaction (PCR) amplification followed the protocol published by Hu et al. [60] with modifications. It was conducted on an ABI GeneAmp PCR system 9700 (LT, Applied Biosystems/Life Technologies, Paisley, UK) thermal cycler with a total reaction volume of 10 µl containing 5 µl TaqMan Universal PCR Master Mix (LT), 2.5 µg non-acetylated bovine serum albumin, 4% (v/v) dimethyl sulfoxide (both Sigma-Aldrich, St. Louis, MO, USA),, 100 nM primer HTTLPR_F: GCAACCTCCCAGCAACTCCCTGTA, 100 nM primer HTTLPR_R: GAGGTGCAGGGGGATGCTGGAA (both Microsynth, Balgach, Switzerland), and 2 µl sample DNA. The thermal cycling protocol implied an initial denaturation step at 95°C for 10 min, 40 cycles of 96°C for 15 s and 62.5°C for 90 s, and a final extension step at 62.5°C for 30 min. Laser induced fluorescence capillary electrophoretic separation of the PCR products was performed on an ABI Prism 3100 Genetic Analyzer using POP6, 36 cm capillary arrays (all LT), and default instrument settings. Prior to electrophoresis, 2 µl PCR product were heat-denatured in 20 µl deionised formamide. Amplicon-length determination was based on the GeneScan-500 LIZ internal size standard (LT). Raw data were recorded and analyzed with the ABI Prism 3100 Genetic Analyzer data collection (v 1.1) and the Genotyper (v 3.6 NT) softwares (both LT). For the computer-assisted calling of the 5-HTTLPR s and l alleles, size bins of 131.2±0.5 nt and 175.2±0.5 nt, respectively, were used. Further, a peak height threshold of 250 rfu was defined (typical peakheights obtained for both homozygous states were at about 8500 rfu and those found for heterozygotes fell in the range of 6500–8300 rfu).

#### Genotyping of COMT Val158Met (rs4680)

For the molecular genetic analysis of the human COMT Val158Met polymorphism the high resolution melting (HRM) curve profiles of short PCR amplicons were analysed.

PCR amplification and subsequent HRM analysis were conducted in a homogeneous assay format on a Rotor-Gene Q real-time PCR cycler (Qiagen, Hilden, Germany). The amplification cocktail had a total volume of 20 µl and consisted of 1x Type-it HRM PCR master mix (Qiagen), 250 nM primer COMT-F_−44_ (5′-CCGACTGTGCCGCCATCAC-3′), 250 nM primer COMT-R_+27_ (5′-GTCAGGCATGCACACCTTGTCCTTCA-3′, both Microsynth,; [61]), 5 µg non-acetylated bovine serum albumin, and 2 µl DNA extract.

The cycler protocol used for amplification comprised an initial denaturation step at 95°C for 5 min followed by 40 cycles of 95°C for 15 s and 68°C for 45 s. The final extension step (68°C) was extended by 10 min. To identify samples featuring exceptionally low or high template molecule concentrations and/or aberrant amplification plots, PCR kinetics was determined by the on-line recording of the signal emitted by the bound double-stranded DNA specific EvaGreen fluorescent dye. Following amplification, the PCR products (71 bp) were subjected to a temperature ramp from 68°C to 95°C (0.1°C increments) without any prior manipulations. EvaGreen fluorescence was continuously recorded to monitor DNA strand dissociation kinetics. For the automated COMT Val158Met genotype calling from the HRM data of unknown samples, the Rotor-Gene ScreenClust HRM software (v 1.10.1.2, Qiagen; [62]) was run in the supervised analysis mode. The dissociation curves obtained for a set of samples with known COMT Val158Met genotypes were used as controls for the underlying linear discriminant analysis.

In initial experiments, by this approach 188 concordant genotype inferences were obtained for a set of 189 samples with known rs4680 genotype. Due to an additional sequence variant (Y) in the amplicon, a single sample was erroneously attributed by the ScreenClust software to the A/A (Met/Met) instead of the actual G/A (Val/Met) genotype. However, this particular rs4680 genotype was tagged potentially problematic, as it featured low class membership indices (data not shown).

### Statistical Analysis

The relationships between the genotypes and the two MPT test scores were assessed using two two-way analyses of variance, using the COMT genotype and the 5-HTTLPR genotype as between-subjects factors, and either CR or SR as the dependent variable. The effects of the two genotypes were simultaneously analyzed in one analysis, in order to be able to test the effect of the COMT genotype independently of the 5-HTTLPR genotype and vice versa (using the regression approach to the analysis of variance, which also controls for unequal cell sizes). In addition, analysis of covariance was conducted to test whether any observed associations for one of the MPT scores remained significant after adjusting for the other, that is, if the effects were either due to shared or unique variance of the two MPT measures.

## Results

The analysis with CR as the dependent variable yielded a significant main effect of COMT genotype (*F*_2,246_ = 4.4, *p* = .013, *η_p_* ^2^ = .034). There was no significant main effect of 5-HTTLPR (*F*_2,246_ = 1.82, ns.; interaction effect COMT by 5-HTTLPR *F*_4,246_ = .68, ns.). By contrast, in the analysis with SR as the dependent variable, the main effect of 5-HTTLPR was significant (*F*_2,246_ = 3.58, *p* = .029, *η_p_^2^* = .028), whereas no significant effect was observed for COMT (*F*_2,246_ = 1.62, ns.; COMT by 5-HTTLPR *F*_4,246_ = .51, ns). Means and standard deviations for the main effects of COMT and 5-HTTLPR are shown in Table 2.

**Table 2:** Effects of COMT and 5-HTTLPR on the inhibition (CR) and memory updating (SR) components of the MPT.

	COMT			5-HTTLPR		
	Val/Val	Val/Met	Met/Met	s/s	s/l	l/l
	(*n* = 51)	(*n* = 124)	(*n* = 80)	(*n* = 90)	(*n* = 106)	(*n* = 59)
CR	.227	.211	.241	.241	.219	.220
	(.062)	(.056)	(.062)	(.085)	(.070)	(.061)
SR	.037	.031	.037	.042	.029	.033
	(.027)	(.026)	(.033)	(.030)	(.029)	(.027)

*Note*. Means (standard deviations). The main effect of COMT is significant for CR but is not significant for SR. The main effect of 5-HTTLPR is significant for SR but is not significant for CR.

Post-hoc tests (Tukey's HSD) indicated that homozygous Met carriers exhibited poorer performance in the inhibition component of the MPT (CR) than Val/Met carriers, whereas those homozygous for the Val allele did not show any further improvement, thus suggesting that the inhibition of developing routines is impaired in individuals homozygous for the Met allele compared with those carrying one or two copies of the Val allele. Similarly, individuals homozygous for the 5-HTTLPR s-allele showed poorer performance on the memory component of the MPT (CR) than s/l carriers, with no further improvement in l/l homozygotes, suggesting poorer memory monitoring and updating skills in individuals homozygous for the s-allele compared with carriers of the l-allele. To confirm these conclusions, the analyses were repeated testing the COMT Met/Met genotype against the combined Met/Val and Val/Val group, and testing the 5-HTTLPR s/s genotype against the combined s/l and l/l group. The patterns of findings were confirmed for both MPT measures. The significant main effect of COMT for CR showed impaired inhibition in individuals homozygous for the Met allele (*M* = 0.247, *SD* = 0.091) as compared to carriers of at least one Val allele (*M* = 0.218, *SD* = 0.058; *F*_1,251_ = 6.87, *p* = .009, *η_p_^2^* = .027; main effect of 5-HTTLPR *F*_1,251_ = 4.18, *p* = .042, *η_p_^2^* = .016; interaction effect COMT by 5-HTTLPR *F*_1,251_ = 0.25, ns.). The significant main effect of 5-HTTLPR for SR showed impaired memory updating in homozygous s/s carriers (*M* = 0.043, *SD* = 0.030) as compared to those carrying at least one long allele (*M* = 0.031, *SD* = 0.028; *F*_1,251_ = 7.50, *p* = .007, *η_p_^2^* = .029; COMT *F*_1,251_ = 1.39, ns.; COMT by 5-HTTLPR *F*_1,251_ = 0.17, ns.).

**Figure 1:**
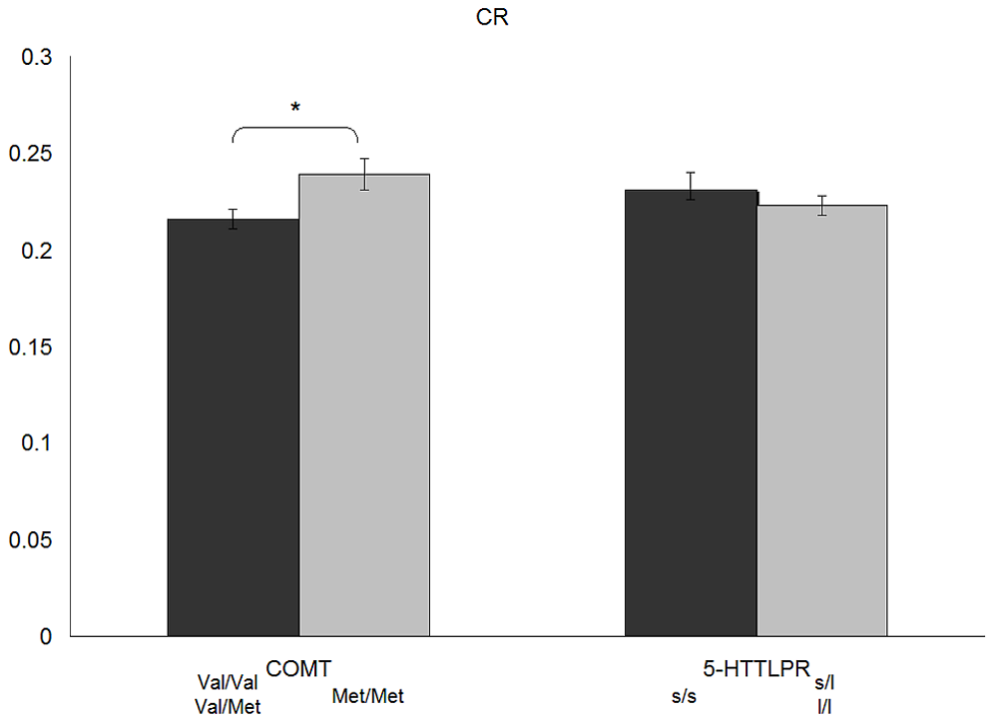
Effect of COMT-polymorphism and 5-HTTLPR on the Context Redundancy (CR) of the MTP.

**Figure 2:**
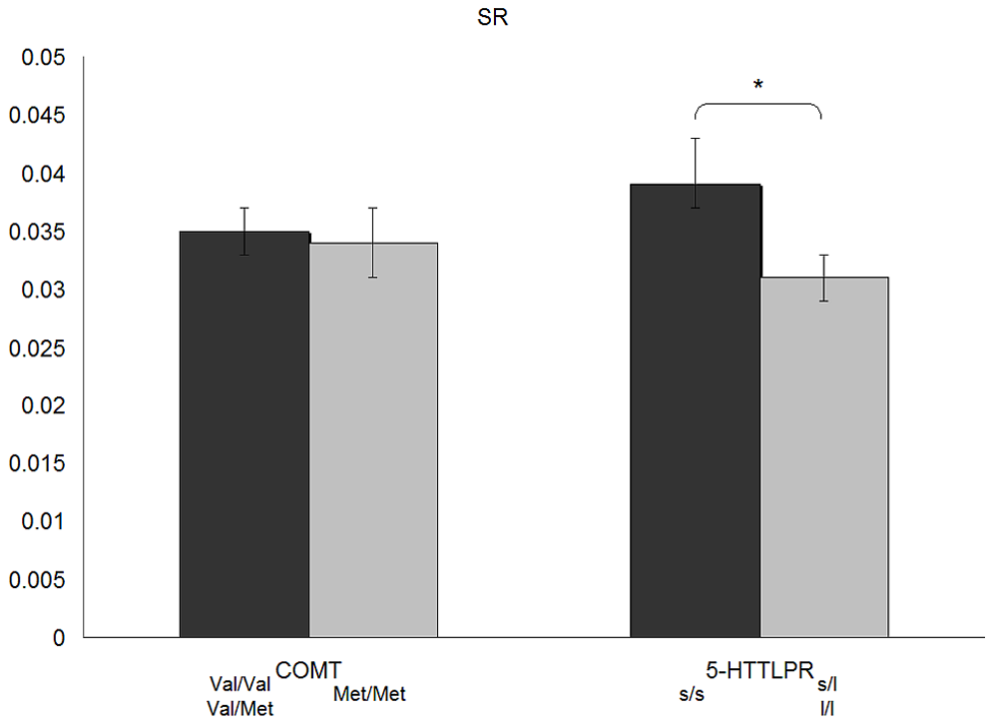
Effect of COMT-polymorphism and 5-HTTLPR on the Symbol Redundancy (SR) of the MTP.

Confirming the double dissociation pattern, both the effects of COMT on CR (COMT *F*_1,250_ = 5.44, *p* = .020, *η_p_^2^* = .021; 5-HTTLPR *F*_1,250_ = 0.65, ns.; COMT by 5-HTTLPR *F*_1,250_ = 1.17, ns.) and the effect of 5-HTTLPR on SR (5-HTTLPR *F*_1,250_ = 3.90, *p* = .049, *η_p_^2^* = .015; COMT *F*_1,250_ = 0.01, ns.; COMT by 5-HTTLPR *F*_1,250_ = 0.03, ns.) remained significant after adjusting for the other MPT variable in analyses of covariance. The means for the main effects of these analyses (adjusted for the other MPT variable) are shown in Figures 1 and 2.

Neither of the two dependent measures were correlated with age (CR: *r* = .01, ns.; SR: *r* = .01, ns.).

## Discussion

Based on the idea that the quantification of executive functions, particularly of indicators of cognitive flexibility, can be improved by considering that they are rooted in the temporal domain [63], the present study analyzed the sequential pattern of responses in a motor random generation task. Using information theoretical measures providing independent indicators of highly specific executive functions in healthy individuals, the study demonstrated differentiated influences of the dopamine-related COMT genotype and the serotonin-related 5-HTTLPR genotype on the inhibition of prepotent responses and the memory updating component of cognitive flexibility.

Consistent with the hypothesis that the COMT polymorphism predicts performance on parameters that have been used to operationalize cognitive flexibility, subjects homozygous for the Met allele showed higher context redundancy in the MPT. Context redundancy reflects the extent to which responses are continuously influenced by previously chosen alternatives and is an indicator of the inefficiency of inhibitory processes, specifically of the inhibition of developing routines. Therefore, homozygosity for the Met allele was related to poorer inhibition and greater stereotypy compared to the Met/Val and Val/Val genotypes. Our observation that the COMT polymorphism modulated the inhibition component of cognitive flexibility provides some support for the tonic/phasic dopamine hypothesis [64]–[65]. The tonic/phasic dopamine hypothesis suggests that the Met allele is associated with high tonic dopamine levels in the prefrontal cortex (PFC), which is beneficial for cognitive stability but reduces cognitive flexibility, while on the other hand the Val allele is associated with low tonic and high phasic dopamine levels in PFC, thus enhancing cognitive flexibility but impairing cognitive stability [2]; [66]. Accordingly, previous studies showed an advantage of the Met allele in tasks demanding cognitive stability using the Competing Program Task [19] or the Stroop paradigm [13], which measure mainly cognitive control or conflict processing, but higher cognitive flexibility in participants with one or two copies of the Val allele compared to Met/Met homozygous individuals [18]. The present study adds to this evidence by demonstrating this relationship with a highly specific measure of cognitive flexibility.

Previous studies using the MPT in clinical samples such as schizophrenic patients. consistently showed that schizophrenic patients have higher context redundancy in the MPT (i.e., worse performance in the inhibition component of random motor generation) than healthy controls [36]–[38]; [67] as well as neurotic patients and patients with major depression [34]–[36]. No differences between schizophrenia patients and healthy and other clinical groups were found in the memory-related MPT measure SR [36]. In healthy samples, the MPT measures CR and SR are uncorrelated or only weakly correlated [36]; [48]; [68]–[69]. In line with this finding, in the present study the effects of the genotypes on CR and SR remained significant after adusting for the other MPT variable, confirming that non-overlapping variance of CR and SR accounted for the relationships to the COMT and 5-HTTLPR genotypes, respectively.

In the current study, the analysis of the 5-HTTLPR genotype showed that individuals homozygous for the s-allele produced higher symbol redundancy in the MPT. Symbol redundancy refers to a balanced selection of single keys and is related to the memory-updating component of random sequence generation [32]. Thus, higher symbol redundancy in individuals homozygous for the s-allele points to a restricted ability to monitor and update information in working memory. This result corresponds well with recent findings of poorer performance in s-allele carriers for certain aspects of memory in older adults [70]–[72]. Pacheco et al. [70] found specific deficits in memory monitoring in a source recognition memory task accompanied by less neural activity in regions of the prefrontal cortex in older adults who carried at least one copy of the s-allele. But also in young adults, Price et al., [30] could show that the S allele may be associated with reduced 5-HT signaling, thus increasing the risk for subtle hippocampal and memory abnormalities. However, the association between 5-HTTLPR and impaired figural memory performance as well as larger left hippocampal volume was only found in healthy young women, but not in men, suggesting that gender may moderate the functional effects of 5-HTTLPR on neurocognition. Also in line with the present findings and the well-established association between the ss genotype and depression [73], treatment with a selective serotonin reuptake inhibitor improved temporal order memory performance [74]. In rats, prefrontal serotonin depletion was specifically related to working memory performance relying on egocentric information (i.e., their own behavior) but not to performance relying on external sensory information [55], mirroring the demands in the MPT where only internal but not external cues guide the selection of the next response [40].

However, the role of serotonin in cognition is among the least understood of monoaminergic neurotransmission [3]; [75]. Several studies investigating the effect of serotonin level on cognition by using acute tryptophan depletion found impaired performance on a variety of tasks such as episodic memory performance, decision making, as well as reduced sensitivity to reward and a negative emotional bias, but unaffected or even improved executive functions such as attention, working memory, and planning [27]; [76]–[80]. Most studies investigating the influence of 5-HTTLPR on cognitive functions indicated enhanced executive functions in healthy s-allele carriers including several functions involved in working memory [24]–[25]; [27]; [81]–[83], though some studies failed to find significant associations [84]–[85]. Taken as a whole, to date there is little consistent literature regarding the impact of the 5-HTTLPR polymorphism on executive functioning. The present results may provide an important addition to the literature by showing an association of the 5-HTTLPR genotype to a highly specific aspect of memory and executive functioning.

Again, one caveat in linking genes to cognitive functions is the use of diffuse indicators of brain functions such as many standard neuropsychological tests which measure several cognitive components at the same time. Tests such as the MPT may help to disentangle more specific processes as compared to the more classical tests. The MPT has also advantages compared to the more commonly used random number generation task, in which participants are instructed to say the numbers one to nine in a random fashion for a number of trials. Although there are great similarities, some features of the MPT allow more straightforward interpretations of the functionality of the inhibition and the memory components of random sequence generation [48]; [56]. In random number generation (RNG), two inhibitory processes, that is, the inhibition of overlearned response stereotypes (counting) and the permanent inhibition of developing routines are confounded. By contrast, using unlabelled keys that are distributed over the keyboard in an irregular pattern, the CR index in the MPT reflects the latter component only, thus specifically indicating the perseverative repetition of sequences. Similarly, RNG tasks place an additional demand on memory. Whereas in both types of tasks it is important to keep track on the chosen responses (monitor and update the contents of memory), RNG additionally requires to have the pool of candidate choices ready by short-term memory processes, which can be an important source of difficulty in patients [86]. Consequently, the SR index in the MPT is a more specific indicator of memory monitoring and updating performance. The definiteness of the MPT scores, particularly of SR is additionally improved by their independence of preexisting individual preferences for or aversions to certain numbers or letters, which can explain great amounts of variance that may exceed that of differences in memory processes [56]; [87]–[89]. Due to the calculation of the scores, which is based on information theory, the average values of the MPT scores, particularly of SR, may seem very low. However, there is nevertheless substantial variability in the data. Several studies demonstrating that significant parts of the variability of SR and CR are explained by individual differences in the efficiency of executive processes in healthy individuals [32], [42]–[46] as well as demonstrating differences between healthy individuals and patients with relevant psychiatric and neurological disorders such as schizophrenia and Parkinson’s disease [34]–[41] confirmed that this variability is meaningful. Retest correlations (temporal stability) of SR and CR are in a range that is common for behavioral tests [48].

A limitation of the study is that the statistical effects of the genetic variants are only small. However, specific genetic variants typically only account for very small amounts of variance in psychological traits. Even when all variants for which evidence suggests an effect are aggregated, the total variance explained typically does not exceed a few percent [90]. The most obvious cause for the small statistical effects is that many genes contribute to the production of a given psychological trait, so that the variation in any single contributing gene will necessarily produce only a very small effect. Additional explanations include epigenetic effects, epistasis, and gene-environment interactions [90]. In addition, the sample was all female and all Caucasian, which may raise the issue of generalizability of the findings and points to the need for replication. We decided to use a female-only sample for the present study, because sex differences in the genetic architecture of many human traits, psychiatric phenotypes and brain functions, are well addressed in the literature and accumulating evidence show that COMT has markedly sexually dimorphic effects on brain function and its dysfunction in psychiatric disorders (for a review see [91]). Additionally, converging lines of evidence suggest that gender may also moderate the functional effects of 5-HTTLPR on memory [39], decision making [92] and negative affect [93]. In the current study, we did not have menstrual cycle data on the female participants, and one important direction for future research is the inclusion of such data, since previous studies reported about an interacting effect of the hormonal status and genetic polymorphisms [94] as well as fluctuations of the dependent variables across the menstrual cycle [43].

Further, in the present study, relatively poorer performance was observed in only one group of the COMT and 5-HTTLPR polymorphisms, respectively (Met/Met and s/s). Val/Val homozygotes and l/l homozygotes did not show any further performance improvements as compared to the respective heterozygous group. The literature on genetic effects on cognition is mixed regarding the most impaired groups, and demonstrated effects have rarely been linear. In the present study, a possible explanation for the failure to show more linear effects in the data may be ceiling effects in the relatively young and healthy sample, so that further improvement may have been hardly possible. Nevertheless, replication in further studies seems particularly important for the effect of 5-HTTLPR on memory updating, which is less obvious than the COMT effect on prepotent response inhibition.

Taken together, the present results indicate that dopaminergic and serotonergic genes may differentially modulate important and definite executive sub-processes in healthy adult women. Whereas the Val-allele of the COMT polymorphism was associated with better performance on the inhibition component of cognitive flexibility, the l-allele of the 5-HTTLPR genotype was associated with better memory monitoring and updating.
